# Intratumoral microbiota-host gene associations characterize stage-associated microecological patterns in colorectal cancer

**DOI:** 10.3389/fmicb.2026.1934580

**Published:** 2026-09-28

**Authors:** Zhiyuan Chen, Wenxuan Gan, Fang Cao, Fan Yu, Wanling Lin, Xinyue Wang, Yiqun Hong, Meiling Yang, Linjie Hong, Congjie Chen, Jing Wu, Xianzhi Liu, Weijing Zhang, Yifan Lian

**Affiliations:** 1 Department of Gastroenterology, Xiang'an Hospital of Xiamen University, School of Medicine, Xiamen University, Xiamen, Fujian, China; 2 Central Laboratory, The Second Affiliated Hospital of Fujian Medical University, Quanzhou, Fujian, China; 3 Department of Gastroenterology, The Second Affiliated Hospital of Fujian Medical University, Quanzhou, Fujian, China; 4 Department of Radiology, State Key Laboratory of Oncology in South China, Guangdong Provincial Clinical Research Center for Cancer, Sun Yat-sen University Cancer Center, Guangzhou, Guangdong, China; 5 Department of Gastroenterology, Zhongshan Hospital of Xiamen University, Xiamen, Fujian, China

**Keywords:** colorectal cancer, disease stage, intratumoral microbiota, microbe-host association, multi-omics integration

## Abstract

**Introduction:**

Intratumoral microbiota are emerging as important ecological components of the colorectal cancer (CRC) microenvironment. However, their stage–associated patterns and coordination with host gene expression remain poorly understood. This study aimed to characterize stage–associated associations between intratumoral microbiota and the host transcriptome in CRC.

**Methods:**

We profiled 73 regional tumor specimens from 30 independent, treatment-naïve CRC patients using full-length 16S rRNA gene sequencing and mRNA sequencing. Microbial diversity, predicted functional profiles, co-occurrence networks, differential abundance, and microbiota–host transcriptome associations were analyzed. Regularized canonical correlation analysis and machine-learning modeling with SHAP-based feature interpretation were performed to identify stage-discriminative features. Candidate associations were evaluated in two independent public microbiome datasets. Mendelian randomization and immune-infiltration analyses were further conducted to explore the potential biological relevance of representative microbiota–gene associations.

**Results:**

Disease stage was a major factor shaping intratumoral microbial composition. Advanced-stage CRC exhibited increased microbial α-diversity, distinct predicted functional profiles, and altered microbial co-occurrence patterns. Integrated microbiota–transcriptome analysis identified ten genus-level and six species-level candidate microbiota–gene association pairs. Core genera showed reproducible stage-discriminative signals in two independent public microbiome datasets. SHAP analysis identified the principal microbial and host transcriptomic features contributing to stage classification. Integrating microbial and host gene signatures improved classification performance, with the combined model achieving an AUROC greater than 0.90. The *Alloprevotella*–MDH1 pair was selected as a representative association axis. *Alloprevotella* abundance was negatively correlated with MDH1 expression, while low MDH1 expression was associated with poorer survival and distinct metabolic–immune states in CRC. Exploratory Mendelian randomization analysis provided preliminary genetic epidemiological support for an association between *Alloprevotella* abundance and CRC risk.

**Discussion:**

Intratumoral microbiota and host transcriptional profiles exhibit coordinated, stage-associated alterations in CRC. The identified microbiota–gene associations, particularly the *Alloprevotella*–MDH1 axis, provide potential biological links between tumor microbial ecology, host metabolism, immune states, and CRC progression. These findings support the value of integrated microbiome–transcriptome analysis for characterizing the intratumoral microenvironment and identifying candidate microbial–host regulatory relationships.

## Highlights

- Intratumoral microbiota show stage-associated ecological differences, characterized by increased diversity, altered composition, and exploratory co-occurrence patterns.
- Integrated microbiome-transcriptome analysis identifies candidate microbe-host gene modules associated with metabolism, immune regulation, tumor signaling, and stage-related biological stratification.
- The *Alloprevotella*-MDH1 association emerges as a representative microbe-host link associated with survival-related features, metabolic-immune programs, immune infiltration patterns, and potential disease risk.

## 1 Introduction

CRC is one of the most common malignancies worldwide and remains a leading cause of cancer-related morbidity and mortality (Sung et al., 2021). Despite substantial advances in screening, surgical management, and systemic therapies, disease stage at diagnosis continues to be a major determinant of treatment strategy and clinical outcome (Dekker et al., 2019). In general, patients with early-stage CRC can achieve relatively favorable survival after curative treatment, whereas those with advanced-stage disease are more likely to develop lymph node involvement, distant metastasis, treatment resistance, and poor prognosis. Therefore, the biological and ecological differences that distinguish localized from advanced CRC represent a major clinical and scientific concern. Although the current TNM staging system provides an effective framework for anatomical classification and therapeutic decision-making, it does not fully explain the molecular and ecological heterogeneity observed across different disease stages (Guinney et al., 2015; Dienstmann et al., 2017a). In particular, the local ecological factors associated with early- and advanced-stage CRC remain incompletely understood.

Accumulating evidence indicates that microbiota-related ecological alterations are important environmental factors involved in colorectal tumorigenesis and progression (Schwabe and Jobin, 2013; Wong and Yu, 2019; Si et al., 2021). Specific microbial taxa have been implicated in CRC development through multiple mechanisms, including induction of chronic inflammation, disruption of epithelial barrier integrity, modulation of host metabolism, interference with antitumor immunity, and enhancement of genomic instability (Louis et al., 2014; Gur et al., 2015; Chung et al., 2018; Pleguezuelos-Manzano et al., 2020). Beyond fecal microbial communities, increasing attention has been directed toward the local microbial ecosystem within tumor tissues, as tumor-associated microorganisms may more directly reflect the biological processes occurring in the tumor microenvironment (Nejman et al., 2020). Emerging studies suggest that intratumoral microbiota is associated with host transcriptional states, immune contexture, metabolic adaptation, and tumor heterogeneity (Shi et al., 2025; Cao et al., 2024).

Recent multi-region and spatial studies have further demonstrated substantial microbial heterogeneity among anatomically distinct regions of the same colorectal tumor. Liu et al. reported that separate biopsies from the same colorectal neoplasm could display different microbial profiles (Liu et al., 2021), whereas Galeano Niño et al. showed that bacteria-enriched and bacteria-poor regions could coexist within an individual tumor (Galeano Niño et al., 2022). These findings indicate that a single-region specimen may incompletely represent the intratumoral microbial community and provide a rationale for multi-region sampling. Recent reviews have also emphasized the bidirectional nature of microbiota-cancer relationships (Moshref-Javadi et al., 2026), while computational pathology studies have suggested that microbiome-related tissue features may vary according to pathological stage and anatomical site, although such computationally inferred signatures should be distinguished from direct microbial measurements (Steele et al., 2026).

However, most previous studies in CRC have focused on comparisons between tumors and healthy controls, or on fecal microbial markers for early detection and non-invasive diagnosis (Dai et al., 2018; Wu et al., 2021; Yu et al., 2017a; Baxter et al., 2016). By comparison, the compositional characteristics, functional remodeling, and host-coupled features of intratumoral microbiota across different clinical stages of CRC remain poorly characterized. In particular, it is still unclear whether early- and advanced-stage CRC harbor distinct tumor-associated microbial patterns and whether these features are linked to host gene expression programs.

In this study, we performed an integrated analysis of intratumoral microbiota and host transcriptomic profiles in multi-region primary tumor specimens from treatment-naïve patients with early- and advanced-stage CRC. Using 16S rRNA sequencing, transcriptomic profiling, multi-omics integration, and machine-learning approaches, we systematically characterized stage-associated alterations in microbial composition, computationally inferred microbial functions, and statistical relationships between microbial taxa and host transcriptional profiles. We hypothesized that early- and advanced-stage CRC exhibit distinguishable intratumoral microbial patterns and that these patterns are associated with host transcriptional programs. By integrating intratumoral microbiome profiling with host transcriptomic analysis, this study provides a tissue-level view of the ecological and molecular changes between the two stage groups. The identified microbial taxa and microbe-host associations may provide candidate features for exploratory biological stratification and testable hypotheses for subsequent mechanistic investigation.

## 2 Materials and methods

### 2.1 Study population and clinical definitions

Patients with pathologically confirmed CRC who underwent surgical resection at Zhongshan Hospital of Xiamen University were enrolled in this study. All included patients were newly diagnosed and had received neither anticancer therapy nor therapeutic antibiotic treatment before the day of surgery. All patients underwent the same standardized perioperative protocol, including preoperative bowel preparation with polyethylene glycol and intravenous prophylactic antibiotic administration approximately 30 min before surgery and continued for 48 h postoperatively. Patients with incomplete clinicopathological information or unavailable pathological staging data were excluded. Regional specimens that failed either DNA- or RNA-based quality control were excluded from both analyses. All analyzed specimens were obtained from primary tumors rather than metastatic lesions.

Clinical information, including age, sex, body mass index (BMI), tumor size, specific primary tumor location, individual T, N, and M categories, lymph-node metastasis status, distant metastatic status, smoking history, and alcohol consumption history, was collected from medical records. Disease stage was defined according to the postoperative pathological TNM (pTNM) classification: stage I was defined as T1–T2N0M0, stage II as T3–T4N0M0, stage III as any T with regional lymph-node metastasis (N1–N2) but no distant metastasis (M0), and stage IV as any T and any N with distant metastasis (M1). Stage I/II CRC was defined as early-stage disease, whereas stage III/IV CRC was defined as advanced-stage disease. The decision to proceed with upfront surgery was based on the preoperative clinical assessment, whereas final disease stage was determined according to postoperative pathological findings. Accordingly, some patients were classified as having advanced disease only after pathological examination revealed regional lymph-node involvement. Although neoadjuvant treatment is generally considered for clinically locally advanced rectal cancer, the patients with pStage IV rectal cancer included in this study underwent upfront palliative surgery because of substantial physical symptoms.

Patient-level covariate balance across disease-stage groups was evaluated using standardized mean differences (SMDs). The assessed variables included age, sex, BMI, primary tumor laterality (left vs. right), primary tumor site (colon vs. rectum), tumor size, smoking history, and alcohol consumption. All participants were fully informed about the study and agreed to participate. This study was conducted in accordance with the Declaration of Helsinki and was approved by the Clinical Research Ethics Committee of Zhongshan Hospital Xiamen University (approval no. xmzsyyky-2023-111).

### 2.2 Multi-region tumor sampling

To characterize intratumoral microbial spatial heterogeneity and reduce potential bias arising from single-region sampling, a multi-region tumor sampling strategy was used. Immediately after surgical resection, 2–3 spatially distinct regions of each primary tumor were sampled within the sterile operating-room field using sterile instruments, while avoiding contact with intestinal contents and non-sterile surfaces. Each specimen was transferred to a sterile labeled tube, snap-frozen in liquid nitrogen, and stored at −80 °C until nucleic acid extraction.

Two unused tubes from the same batch were included as contemporaneous blank controls. Except for the absence of tumor tissue, these controls underwent the same operating-room exposure, handling, transportation, DNA extraction, and pre-library quality assessment as the biological specimens. The quality-control results indicated minimal background contamination, supporting the reliability of the sampling and processing procedures.

All candidate regional tumor specimens subsequently underwent quality-control assessment for both DNA-based microbiome analysis and RNA-based host-transcriptome analysis. Specimens failing quality control for either assay were excluded from both analyses; only specimens that passed both quality-control criteria were retained. The patient was defined as the independent biological unit, whereas regional specimens were treated as repeated observations nested within patients. Depending on the analytical method, analyses were conducted at either the patient level or the regional-specimen level, with patient ID incorporated where appropriate. The specific procedures are described in the corresponding sections below.

### 2.3 Microbial DNA extraction and 16S rRNA gene sequencing

Microbial DNA was extracted from frozen tumor tissue samples using a standardized DNA extraction protocol suitable for tissue-associated microbiome profiling. DNA concentration and purity were assessed before library preparation. To reduce potential contamination in low-biomass tumor tissue samples, sterile instruments, clean consumables, and contamination-control procedures were used during tissue processing, DNA extraction, and PCR preparation.

Full-length 16S rRNA gene sequencing was performed to profile the intratumoral microbiota. Sequencing libraries were generated from bacterial 16S rRNA gene amplicons, followed by purification, quantification, pooling, and high-throughput sequencing according to the sequencing provider's standard protocol. After quality control and filtering, high-quality bacterial sequencing reads were retained for downstream taxonomic profiling.

### 2.4 Processing of 16S rRNA sequencing data

Raw 16S rRNA sequencing reads were subjected to demultiplexing, quality filtering, denoising, and chimera removal using a standardized microbiome profiling pipeline. High-quality bacterial features were retained for downstream analysis. Taxonomic annotation was performed against the Greengenes2 database. Taxonomic abundance tables were generated at the phylum, genus, and species levels.

Relative abundance profiles were used for microbial composition analysis. For compositional beta-diversity analysis, microbial abundance data were transformed using centered log-ratio transformation after adding a pseudocount, and Aitchison distance was calculated.

### 2.5 Microbial diversity and community composition analysis

Alpha-diversity indices from the 73 regional specimens were analyzed using linear mixed-effects models, with disease stage as a fixed effect and patient ID as a random intercept to account for within-patient correlation. Statistical inference for the stage effect was based on the Kenward-Roger approximation for denominator degrees of freedom.

Beta-diversity was assessed based on Aitchison distance. Principal coordinate analysis (PCoA) was used to visualize differences in intratumoral microbial community structure between early- and advanced-stage CRC. Permutational multivariate analysis of variance (PERMANOVA) was performed to evaluate whether disease stage significantly explained variation in microbial composition.

Microbial community composition was summarized at the phylum, genus, and species levels. Dominant taxa were visualized using stacked bar plots and compared between stage I/II and stage III/IV CRC samples.

### 2.6 Association between clinical variables and intratumoral microbiota

Hierarchical association analysis was performed using HAllA to evaluate associations between clinicopathological variables and intratumoral microbial features. Clinical variables included disease stage, age, sex, BMI, tumor size, tumor location, smoking history, alcohol consumption history, and distant metastatic status. Microbial features at genus and/or species levels were included in the association analysis. Regional profiles were retained to preserve the paired spatial information and intratumoral heterogeneity captured by the multi-region sampling design.

The Benjamini-Hochberg method was used to control the false discovery rate. Significant phenotype-microbiota associations were visualized as hierarchical association heatmaps. Disease stage was subsequently used as the primary phenotype of interest for stage-associated microbiome analysis.

### 2.7 Microbial co-occurrence network analysis

To explore microbial co-occurrence patterns within the intratumoral microbiota, SparCC network analysis was performed at the regional-specimen level using the 73 regional tumor specimens. Separate networks were constructed for stage I/II and stage III/IV CRC to preserve the spatial variation captured by the multi-region sampling design. Low-abundance and low-prevalence taxa were removed before network construction. Pairwise correlations among taxa were calculated, and significant correlations were retained for network visualization.

Network topology was visualized using network analysis tools in R. Network characteristics, including node number, edge number, degree, and overall connectivity, were summarized descriptively for each disease-stage group.

### 2.8 Prediction of microbial functional potential

Microbial functional potential was inferred from 16S rRNA gene sequencing data using PICRUSt2 version 2.5.2. KEGG Ortholog abundances were predicted using the default PICRUSt2 pipeline and subsequently aggregated into KEGG pathway abundances. PICRUSt2 pathway comparisons were performed using the 73 regional tumor specimens, with each regional specimen linked to its corresponding patient ID. Differences in predicted pathway abundance between stage I/II and stage III/IV CRC were evaluated using mixed-effects models of the form Pathway abundance ~ Stage + (1 | Patient ID), with disease stage as a fixed effect and patient ID as a random intercept to account for repeated regional measurements within patients. *P* values for the stage effect were adjusted across all tested pathways using the Benjamini-Hochberg procedure to control the false discovery rate. Pathways with significant differences between groups were visualized to characterize stage-associated functional patterns in the intratumoral microbiota.

### 2.9 Differential abundance analysis of microbial taxa

Differential abundance analysis was performed at both the genus and species levels. LEfSe was applied to the 73 regional specimens as an exploratory regional-level screening method to identify taxa showing stage-specific enrichment patterns between stage I/II and stage III/IV CRC. Taxa with a linear discriminant analysis (LDA) score > 2.5 and P < 0.05 were considered as exploratory candidates.

The LEfSe-identified taxa were subsequently evaluated using MaAsLin2. The 73 regional measurements were treated as repeated observations nested within 30 patients, with disease stage included as a fixed effect and patient ID as a random effect to account for within-patient dependence. *P* values were adjusted using the Benjamini-Hochberg procedure, with *q* < 0.10 used as an exploratory FDR threshold. Taxa showing concordant associations in LEfSe and patient-adjusted MaAsLin2 were retained for subsequent microbe-host integration and model construction.

### 2.10 RNA extraction and transcriptomic sequencing

Total RNA was extracted from frozen tumor tissue samples using a standardized RNA extraction protocol. RNA concentration, purity, and integrity were assessed before library construction. Samples passing RNA quality control were used for transcriptomic sequencing.

RNA-seq libraries were prepared using standard library construction procedures and sequenced on a high-throughput sequencing platform according to the manufacturer's protocol. Raw sequencing reads were subjected to quality control, adapter trimming, and low-quality read removal. High-quality reads were processed to obtain gene-level expression profiles for downstream analysis.

### 2.11 RNA-seq data processing and differential expression analysis

Gene expression profiles were normalized before downstream analysis. Genes with low expression across samples were removed. Differential expression analysis was performed using the 73 regional transcriptomic profiles, with each profile linked to its corresponding patient ID to preserve the multi-region information. Disease stage was included as a fixed effect, while patient ID was specified as the blocking variable. Within-patient correlation was estimated using the duplicateCorrelation function and incorporated into the voom precision-weight estimation and the subsequent lmFit model. Differentially expressed genes were identified based on statistical significance and fold-change criteria.

Volcano plots were generated to visualize stage-associated gene expression differences. To evaluate global transcriptomic differences between early- and advanced-stage CRC, PCoA and three-dimensional principal component analysis were performed based on normalized gene expression profiles. PERMANOVA was used to assess whether disease stage explained transcriptomic variation.

### 2.12 Procrustes analysis

To evaluate the global concordance between intratumoral microbial composition and host transcriptomic profiles, Procrustes analysis was performed at the patient level using matched microbiome and transcriptomic profiles. Regional microbial and transcriptomic measurements were linked by patient ID and averaged within each patient to generate 30 matched patient-level profiles. The microbial and transcriptomic ordinations were calculated separately for the CRC I/II and CRC III/IV groups, and Procrustes analyses were performed for each group. The significance of concordance was assessed by patient-level permutation testing within each group.

### 2.13 Sparse canonical correlation analysis

Sparse canonical correlation analysis (sparse-CCA) was performed at the regional-specimen level using matched microbial and host gene-expression profiles from the same 73 regional tumor specimens. Each regional profile was linked to its corresponding patient ID, and regional profiles were retained to preserve the paired spatial information and intratumoral heterogeneity captured by the multi-region sampling design. Genus-level and species-level microbial abundance matrices were analyzed separately with the host gene expression matrix. Sparse-CCA was used as an exploratory multivariate integration approach to identify candidate microbe-host gene modules by projecting microbial and host gene-expression features into a shared latent space and applying LASSO-based sparsity.

Microbial taxa and host genes with non-zero loadings in selected sparse-CCA components were retained. Host genes associated with sparse-CCA components were subjected to KEGG pathway enrichment analysis. Sankey plots were generated to visualize the relationships among microbial features, host gene sets, and enriched biological pathways.

### 2.14 Pathway enrichment analysis and GSVA

KEGG pathway enrichment analysis was performed for host gene sets identified from sparse-CCA components. GSVA was performed at the regional-specimen level using the 73 matched transcriptomic profiles, with each profile linked to its corresponding patient ID. The genes used for pathway-level analysis were derived from the patient-blocked limma-voom differential expression analysis described above. Disease stage was included as a fixed effect, patient ID was specified as the blocking variable, and within-patient correlation was estimated using the duplicateCorrelation function.

Representative enriched pathways identified from genus- and species-level sparse-CCA analyses were selected, and their GSVA scores were compared between stage I/II and stage III/IV CRC samples. Group differences in GSVA scores were evaluated using a mixed-effects framework with disease stage as a fixed effect and patient ID as a random intercept to account for repeated regional measurements within patients. A heatmap was generated to summarize stage-associated pathway activity patterns across tumor samples.

### 2.15 Mantel test

Mantel analysis was performed using matched patient-level profiles. Regional microbial and transcriptomic measurements were linked by patient ID and averaged within each patient. Microbial and transcriptomic distance matrices were then calculated from these patient-level profiles and compared using Mantel tests, with significance assessed by permutation at the patient level. The Mantel correlation coefficient and corresponding *P* value were calculated to evaluate the strength of the microbiome-transcriptome association.

### 2.16 Regularized canonical correlation analysis

Regularized canonical correlation analysis (rCCA) was performed at the regional-specimen level using paired microbial abundance and host gene-expression profiles from the 73 regional tumor specimens, with each regional profile linked to its corresponding patient ID. rCCA was used to identify candidate microbe-host gene associations while reducing overfitting and multicollinearity. Differential microbial taxa and differentially expressed host genes were used as input features, and the analyses were performed separately at the genus and species levels. Microbe-gene pairs with an absolute correlation coefficient > 0.6 were retained and visualized as positive or negative association networks. The principal *Alloprevotella*-MDH1 association was subsequently evaluated at the patient level by averaging the corresponding regional measurements within each patient to generate 30 patient-level profiles, followed by Spearman correlation and stage-adjusted linear regression using the model MDH1 ~ *Alloprevotella* + Stage.

### 2.17 TCGA data acquisition and transcriptomic validation

Transcriptomic and clinical data for CRC were obtained from The Cancer Genome Atlas (TCGA), including TCGA-COAD and TCGA-READ cohorts. Patients were classified into stage I/II and stage III/IV groups according to available TNM staging information.

Expression levels of rCCA-identified host genes were compared between early- and advanced-stage CRC in TCGA. Binary classification models based on genus-level and species-level rCCA-associated host genes were constructed in TCGA to assess whether these host gene features captured stage-related biological differences.

### 2.18 Survival analysis

For survival analysis, host gene signatures derived from genus-level and species-level rCCA results were evaluated in the TCGA CRC cohort. Gene signature scores were calculated based on the expression of selected host genes. Patients were stratified into high- and low-risk groups according to the median signature score. Kaplan-Meier survival curves were generated, and differences in overall survival were assessed using the log-rank test.

For MDH1-specific analysis, TCGA CRC patients were divided into MDH1-high and MDH1-low groups according to the median expression level of MDH1. Kaplan-Meier analysis was used to evaluate the association between MDH1 expression and overall survival.

### 2.19 Machine-learning model construction

Machine-learning models were developed using patient-grouped nested cross-validation, with all regional specimens from the same patient retained in the same fold during both model tuning and evaluation. Inverse cluster-size weights were applied so that each patient contributed equally during model training. Core genus-level microbial features were first used to construct microbiota-only models. Five algorithms were evaluated, including random forest, XGBoost, support vector machine, multilayer perceptron, and naive Bayes.

Model performance was assessed using receiver operating characteristic curves and the area under the receiver operating characteristic curve (AUROC). To evaluate whether host transcriptomic information improved model performance, core genus-level microbial features were integrated with selected host gene features to construct combined microbe-gene models. The performance of microbiota-only and combined models was compared based on AUROC values.

### 2.20 External validation using public microbiome datasets

To assess the generalizability of the core genus model, external validation was performed using two independent public CRC microbiome datasets, Zeller et al. (2014) and Wirbel et al. (2019). Genus-level abundance profiles and clinical stage annotations were extracted from the external datasets. The core genera identified in the discovery cohort were used to evaluate stage-associated classification performance in each external dataset.

ROC analysis was used to assess model performance. SHAP analysis was applied to evaluate whether major contributing genera showed consistent model-explanation patterns across independent cohorts.

### 2.21 SHAP-based model interpretation and interaction analysis

SHAP analysis was used to interpret feature contributions in machine-learning models. For microbiota-only models, SHAP values were calculated to rank the contribution of core microbial genera. For combined microbe-gene models, SHAP values were calculated for both microbial and host gene features.

SHAP interaction analysis was performed to examine interactions between representative microbial features and host gene features. This analysis was used to determine whether model predictions depended on combined microbe-host feature patterns rather than single independent variables.

### 2.22 Gene set enrichment analysis

Gene set enrichment analysis (GSEA) was performed to characterize biological pathways associated with candidate host genes identified from the microbe-host integrative analysis. Samples were stratified according to the expression level of the candidate gene, or genes were ranked according to their correlation with candidate gene expression, depending on the specific analysis. KEGG gene sets were used as pathway references. Pathways with nominal *P* < 0.05 and FDR *q* < 0.25 were considered significantly enriched. Representative enriched pathways were visualized using enrichment plots.

### 2.23 Mendelian randomization analysis

Mendelian randomization (MR) analysis was performed to evaluate the potential causal relationship between candidate microbial taxa and disease risk. Genetic instruments associated with the abundance of candidate microbial taxa were obtained from publicly available microbiome genome-wide association studies. Summary-level GWAS data for the disease outcome were obtained from publicly available GWAS resources.

Single nucleotide polymorphisms associated with the candidate microbial taxa were selected as instrumental variables. Linkage disequilibrium clumping was performed to ensure independence among instruments, and palindromic or ambiguous variants were removed. Instrument strength was assessed using *F* statistics.

The inverse-variance weighted method was used as the primary MR approach, and MR-Egger was used as a complementary method. Sensitivity analyses were performed to evaluate result robustness. Heterogeneity was assessed using Cochran's *Q* test, and horizontal pleiotropy was evaluated using the MR-Egger intercept test. MR estimates were visualized using forest plots and scatter plots.

### 2.24 Immune infiltration analysis

Immune infiltration analysis was performed using bulk transcriptomic data from CRC tumor samples. Immune cell proportions were inferred using a deconvolution-based immune infiltration analysis method.

To evaluate whether the *Alloprevotella*-MDH1 axis corresponded to a specific CRC immune ecological state, samples were divided into high- and low-*Alloprevotella* groups according to the median abundance of *Alloprevotella* and into high- and low-MDH1 groups according to the median expression of MDH1. Immune cell proportions were compared between groups using the Wilcoxon rank-sum test. Results were visualized as boxplots or heatmaps.

### 2.25 Statistical analysis

All statistical analyses were performed using R and Python. Continuous variables were compared using the Wilcoxon rank-sum test or Student's *t* test, depending on data distribution. Categorical variables were compared using the chi-square test or Fisher's exact test. Correlations were assessed using Spearman correlation unless otherwise specified. Multiple testing correction was performed using the Benjamini-Hochberg false discovery rate method. A two-sided *P* < 0.05 was considered statistically significant unless otherwise stated.

Data visualization was performed using R packages for microbiome analysis, multivariate statistics, pathway analysis, survival analysis, and heatmap visualization, as well as Python packages for machine-learning model construction and SHAP-based model interpretation.

## 3 Results

### 3.1 Patient characteristics and study design

This study focuses on stage-associated differences in intratumoral microbial ecology and their statistical associations with the host transcriptome in early- and advanced-stage CRC. The overall study design is illustrated in Figure 1. Briefly, we performed multi-region sampling of early- and advanced-stage CRC tumor tissues and systematically characterized the intratumoral microbiota composition, alpha-diversity, beta-diversity, differential taxa, co-occurrence networks, and computationally predicted functional profiles based on 16S rRNA sequencing. Subsequently, by integrating host transcriptomic data, we analyzed differential gene expression between different clinical stages and evaluated the overall association between intratumoral microbiota structure and host transcriptional profiles. We further employed sparse-CCA, rCCA, Mantel test, GSVA, and machine learning approaches to identify candidate stage-associated core microbiota, host genes, and microbiota-gene association pairs. Finally, we leveraged TCGA and external microbiome datasets to explore the reproducibility, stage-discriminative ability, survival associations, and potential biological relevance of the candidate features.

**Figure 1:**
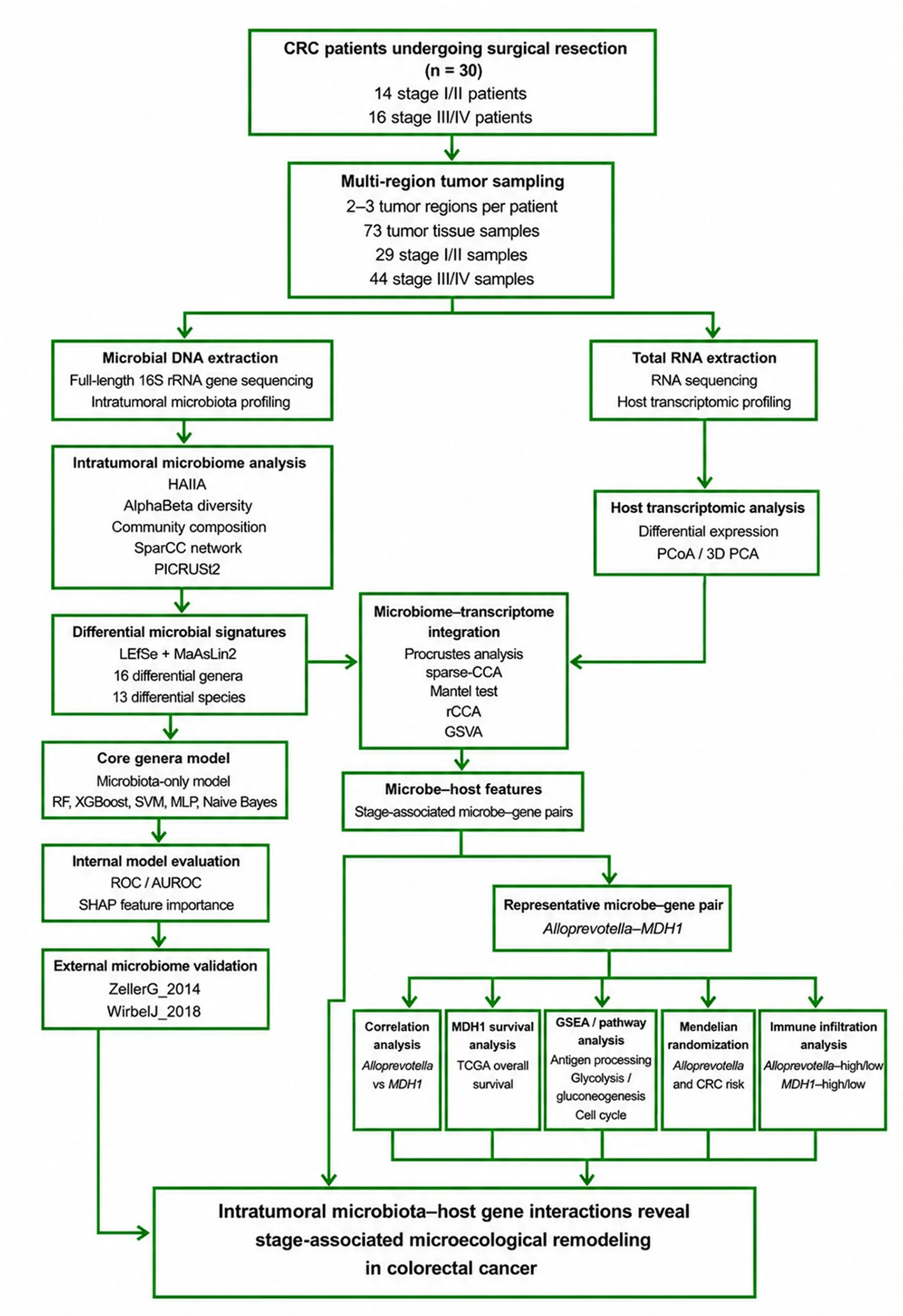
A schematic overview of the study design. Multi-region tumor samples were collected from patients with CRC and classified into stage I/II and stage III/IV groups according to the postoperative pathological TNM (pTNM) classification. Intratumoral microbiota and host transcriptomic profiles were characterized using 16S rRNA gene sequencing and mRNA transcriptome sequencing, respectively. The analytical workflow included microbial diversity analysis, community composition profiling, co-occurrence network construction, functional prediction, differential abundance analysis, transcriptomic profiling, microbiome-transcriptome integration, canonical correlation analysis, machine-learning model construction, external validation, Mendelian randomization, and immune infiltration analysis. The overall aim was to characterize stage-associated intratumoral microecological patterns and their statistical associations with host transcriptional programs in CRC.

In this study, stage I/II CRC was defined as early-stage disease, whereas stage III/IV CRC was defined as advanced-stage disease, according to the postoperative pTNM classification. A total of 30 independent colorectal cancer patients were enrolled in this study, including 14 patients with stage I/II disease and 16 patients with stage III/IV disease. Given that previous studies have demonstrated marked spatial heterogeneity of the microbial community within colorectal tumors and that single-region sampling may not adequately capture the patient-level intratumoral microbiota ecological characteristics (Liu et al., 2021), we adopted a multi-region tumor sampling strategy. During the sampling procedure, 2–3 spatially distinct regions were collected from each primary tumor. 73 spatially distinct regional tumor specimens passed both DNA- and RNA-based quality-control criteria and were retained in the final paired dataset, comprising 29 stage I/II samples and 44 stage III/IV samples. Each patient was considered an independent biological unit, whereas regional specimens were treated as repeated observations nested within patients. Depending on the analytical method, analyses were conducted at either the patient level or the regional-specimen level, with patient ID incorporated where appropriate.

Patient-level balance assessment showed some differences between the disease-stage groups, with the largest differences among non-metastatic variables observed for BMI (absolute SMD = 0.60), primary tumor laterality (0.38), and alcohol consumption (0.39). The remaining covariates showed smaller differences, with absolute SMDs ≤ 0.19. In contrast, distant metastatic status was markedly imbalanced between the two groups (absolute SMD = 0.95). This difference was expected because distant metastasis defines M1 and, consequently, stage IV disease, which was included only in the stage III/IV group. Thus, distant metastatic status is an inherent component of the stage-group definition rather than an incidental baseline difference (Supplementary Figure S1). Detailed patient-level information is provided in Supplementary Table 1, and specific sampling information for patients is presented in Supplementary Table 2.

### 3.2 Stage-associated differences in intratumoral microbiota and predicted microbial functional potential in CRC

To clarify the relationship between clinical factors and intratumoral microbial structure, we first performed an exploratory hierarchical association analysis using HAllA on regional microbial profiles. Among multiple clinical variables, disease stage showed the strongest association with intratumoral microbial composition, indicating that disease stage was closely associated with variation in the local microbial ecosystem (Figure 2A). PCoA based on Aitchison distance further showed a clear separation between stage I/II and stage III/IV CRC samples in microbial composition space (Figure 2B). This stage-associated difference remained evident in the patient-level sensitivity analysis, in which regional microbial profiles were aggregated within each patient and PCoA and PERMANOVA were repeated using 30 independent patient-level profiles (Figure 2C). Alpha-diversity analysis showed that microbial diversity was generally higher in stage III/IV CRC than in stage I/II CRC, with a significant increase in the Shannon index and increasing trends in the Chao1 and ACE indices (Figure 2D). These results indicate that advanced CRC tissues harbor a more complex intratumoral microbial community structure.

**Figure 2:**
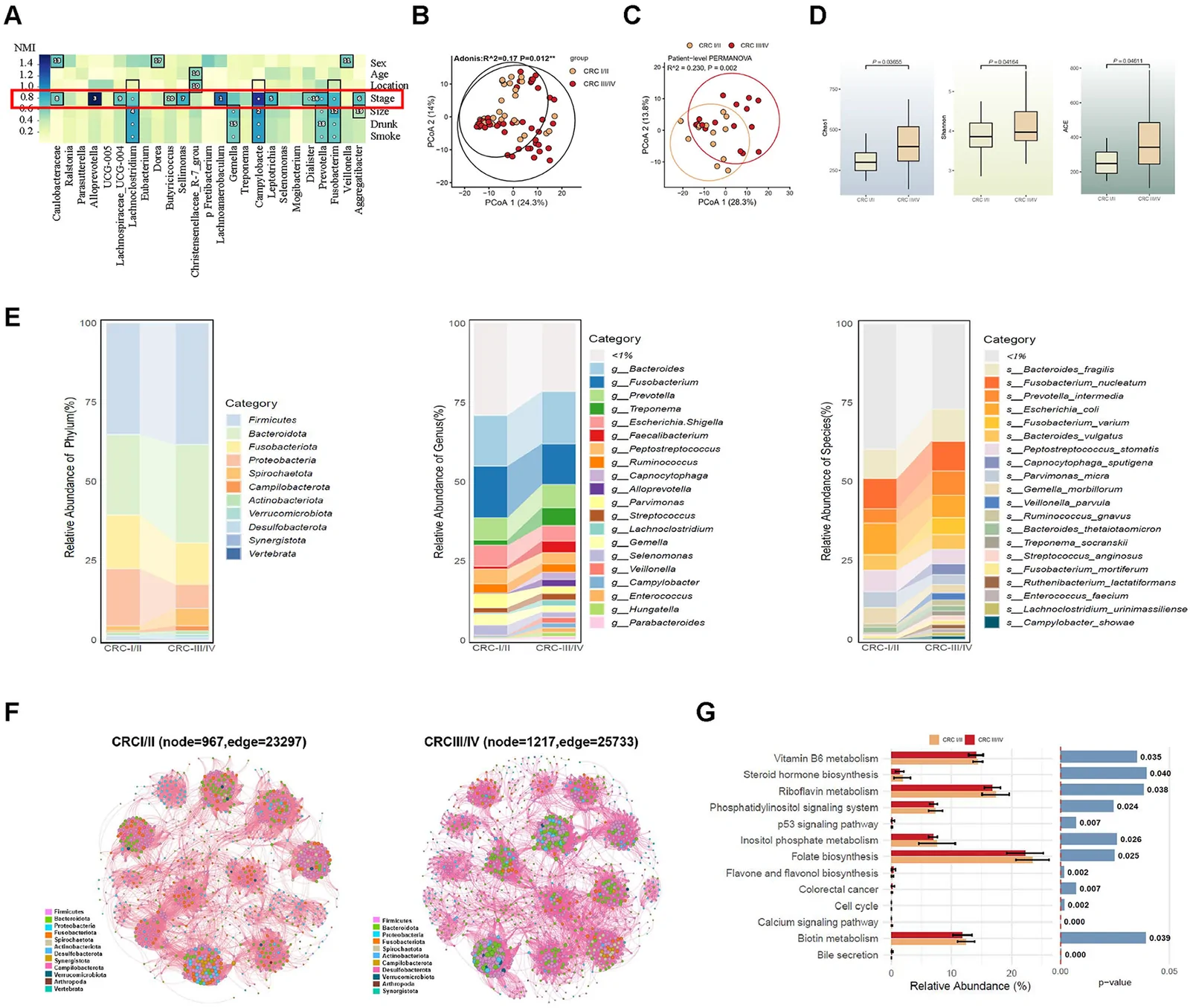
**(A)** Hierarchical association analysis performed using HAllA showing associations between clinicopathological variables and intratumoral microbial features. The heatmap color scale indicates relative pairwise normalized mutual information (NMI). Numbers in cells indicate significant feature pairs ranked according to statistical significance after Benjamini-Hochberg correction, and white dots indicate marginally significant associations. Disease stage was identified as one of the major phenotypic factors associated with intratumoral microbial composition. **(B)** PCoA based on Aitchison distance showing differences in microbial community structure between stage I/II and stage III/IV CRC regional tumor specimens. **(C)** Patient-level PCoA based on Aitchison distance after aggregating regional microbial profiles within each of the 30 patients. Patient-level PERMANOVA showed a significant association between disease stage and microbial community composition. **(D)** Alpha-diversity indices, including Chao1, Shannon, and ACE, comparing microbial richness and evenness between stage I/II and stage III/IV CRC samples. Group differences were assessed using mixed-effects models with disease stage as a fixed effect and patient ID as a random intercept. **(E)** Relative abundance profiles of dominant microbial taxa at the phylum, genus, and species levels in stage I/II and stage III/IV CRC samples. **(F)** SparCC-based microbial co-occurrence networks constructed separately for stage I/II and stage III/IV CRC samples. Nodes represent microbial taxa, and edges represent significant co-occurrence relationships. Network characteristics were summarized descriptively for each disease-stage group. **(G)** Predicted microbial functional profiles inferred using PICRUSt2 based on 16S rRNA gene sequencing data. KEGG Ortholog abundances were predicted and aggregated into KEGG pathway abundances. Differentially enriched KEGG pathways between stage I/II and stage III/IV CRC samples are shown.

At the compositional level, both stage I/II and stage III/IV CRC samples were dominated by *Firmicutes, Bacteroidota, Fusobacteriota*, and *Proteobacteria*, whereas their relative abundance patterns differed between stages. Genus- and species-level analyses further showed that early- and advanced-stage CRC shared a common CRC-associated microbial background but displayed distinct dominant taxa distributions (Figure 2E). Overall, advanced-stage samples showed an increased proportion of microbial groups such as oral-associated bacteria, anaerobes, and opportunistic pathogens, indicating stage-associated differences in the intratumoral microbial composition and local microecological state.

To further assess microbial interactions within the intratumoral ecosystem, we constructed co-occurrence networks using SparCC. The resulting networks were used to visualize co-occurrence patterns in stage I/II and stage III/IV CRC (Figure 2F). PICRUSt2-based functional prediction identified stage-associated differences in microbial functional potential across multiple KEGG pathways, mainly involving microbial metabolism, tumor-associated signal transduction, cell proliferation regulation, and local intestinal ecological functions (Figure 2G). The implicated pathways were related to metabolic adaptation, proliferative processes, and local niche characteristics relevant to advanced CRC.

Together, these results show that stage I/II and stage III/IV CRC differ in intratumoral microbial composition, diversity, microbial co-occurrence patterns, and predicted functional potential, supporting the presence of distinct intratumoral microecological patterns across disease stages.

### 3.3 Identification of stage-associated intratumoral microbial signatures

To further identify stage-associated intratumoral bacterial taxa in CRC, we performed differential abundance analyses at both the genus and species levels. LEfSe analysis showed that stage I/II and stage III/IV CRC samples exhibited distinct enrichment patterns at both taxonomic levels, indicating stage-associated variation in the intratumoral microbial profile (Figure 3A). To improve the robustness of differential taxa identification, we further performed an independent analysis using MaAsLin2. The MaAsLin2 results were largely consistent with the LEfSe findings, further supporting the presence of reproducible intratumoral microbial differences across clinical stages of CRC (Figure 3B).

**Figure 3:**
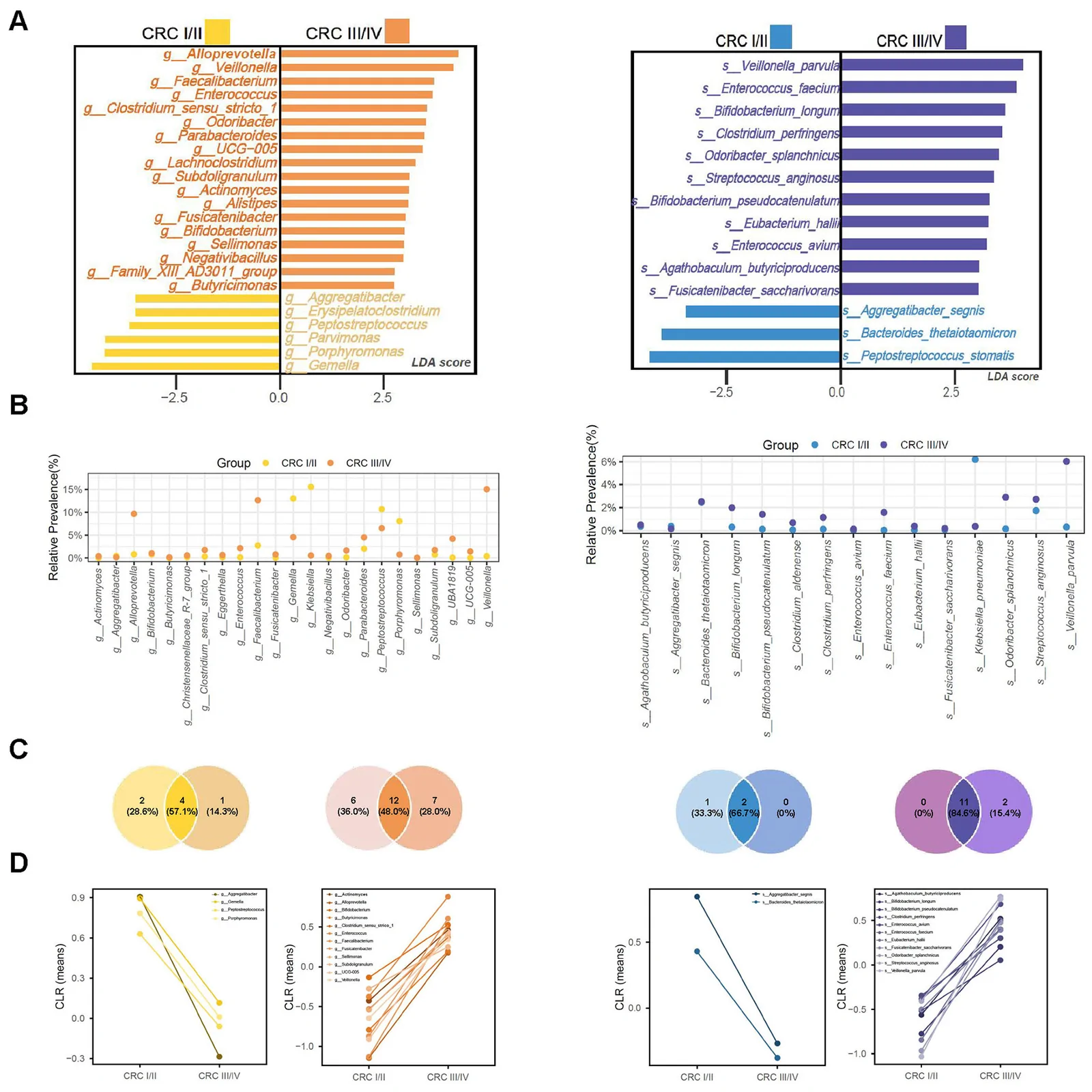
**(A)** LEfSe analysis showing differentially abundant microbial taxa between stage I/II and stage III/IV CRC samples at the genus level and species level. Taxa with LDA score > 2.5 and *P* < 0.05 were considered as exploratory stage-associated candidates. **(B)** Relative abundance of significant microbial taxa subsequently evaluated using MaAsLin2, with disease stage as a fixed effect and patient ID as a random effect. **(C)** Venn diagrams showing the overlap of stage-associated genera and species identified by LEfSe and MaAsLin2. Taxa jointly identified by both approaches were retained as candidate stage-associated microbial features. **(D)** Relative abundance of shared differential microbial taxa between stage I/II and stage III/IV CRC samples.

Based on the differential taxa supported by LEfSe and MaAsLin2, we further evaluated the consistency of stage-associated microbial signatures between the two analytical approaches. Venn diagram analysis showed that overlapping genera and species identified by both methods, which were retained as candidate stage-associated microbial features (Figure 3C). The abundance patterns of these shared differential taxa further illustrated distinct microbial enrichment profiles between stage I/II and stage III/IV CRC samples (Figure 3D). These overlapping taxa covered diverse ecological types, including oral-associated bacteria, anaerobes, opportunistic pathogens, and metabolism-related commensals. Most of them showed increased relative abundance in stage III/IV CRC, suggesting that advanced CRC may be accompanied by more complex remodeling of the intratumoral microbial ecosystem. These consistently supported differential taxa were subsequently used for microbe-host integrative analysis and stage-classification model construction.

### 3.4 Integrated microbiome and transcriptome analyses identify coordinated microbe-host functional patterns across CRC stages

To characterize host transcriptional differences across CRC stages, we next compared gene expression profiles between stage I/II and stage III/IV CRC tissues. Differential expression analysis, PCoA, and three-dimensional principal component analysis consistently showed clear transcriptomic separation between early- and advanced-stage CRC samples, indicating distinct host transcriptional profiles across disease stages (Supplementary Figures S2A–C). After regional microbial and transcriptomic measurements were averaged within each patient, patient-level Procrustes analyses showed significant concordance between the microbial and transcriptomic ordinations in both the CRC I/II group and the CRC III/IV group (Supplementary Figure S2D).

We then applied sparse-CCA to the 73 matched regional microbial and host gene-expression profiles, with each profile linked to its corresponding patient ID, to identify candidate stage-associated microbe-host gene modules. This approach projects microbial abundance profiles and host gene expression profiles into a shared latent space and selects the most correlated microbial and transcriptional features through LASSO-based sparsity. At both the genus and species levels, sparse-CCA identified candidate associations between microbial taxa and host gene sets involved in metabolism, digestive system functions, cellular processes, disease-related pathways, and environmental information processing (Figure 4A). The resulting modules highlighted potential links between intratumoral microbial features and host pathways related to metabolic adaptation, immune regulation, cell adhesion, and tumor-associated signaling.

**Figure 4:**
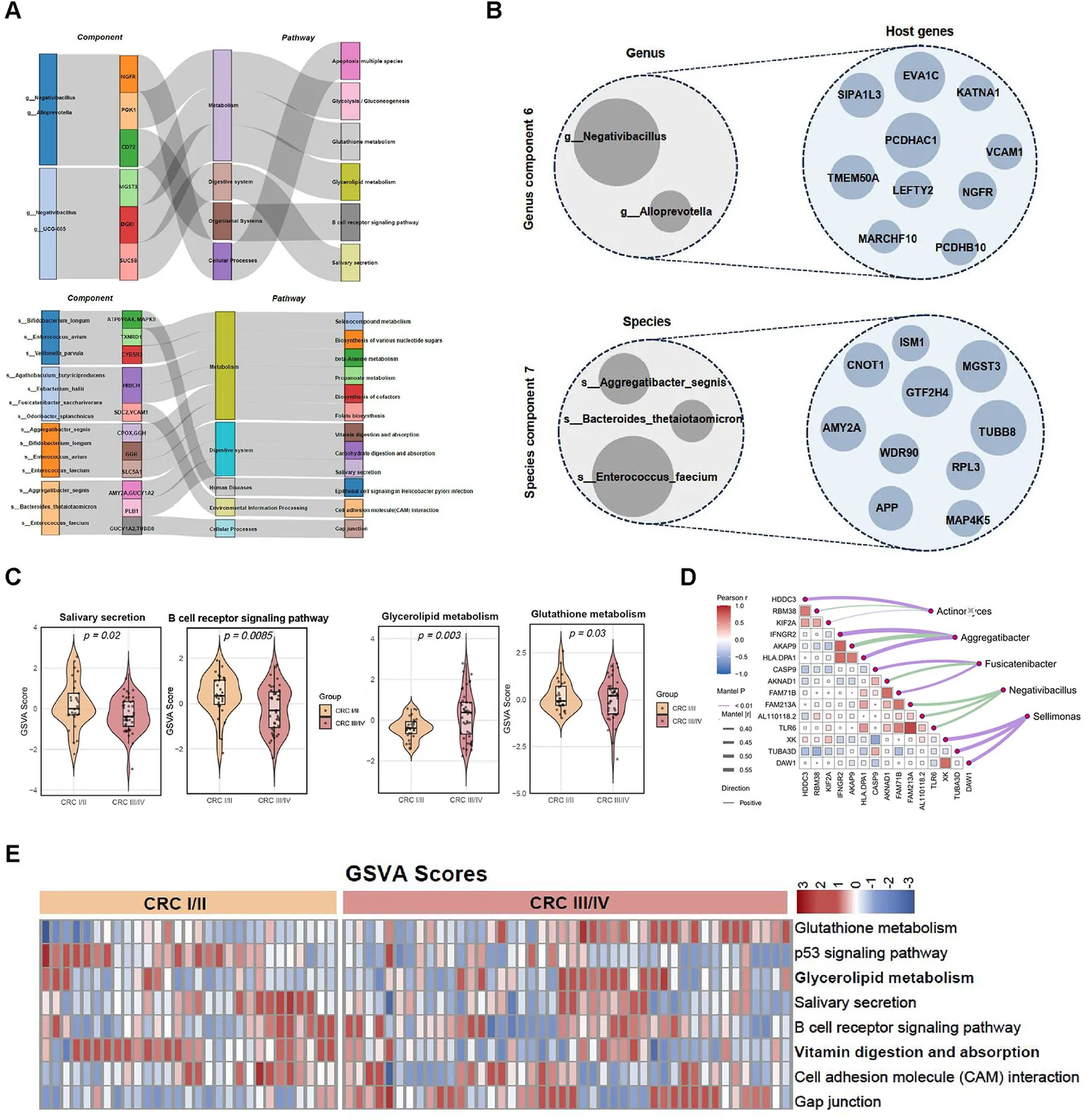
**(A)** Sankey plots showing biological pathways enriched among host genes associated with microbial genera and species identified by sparse-CCA. Sparse-CCA applies LASSO-based feature selection and projects microbial and host gene-expression profiles into a shared latent space to identify correlated microbe-gene components. **(B)** Representative sparse-CCA component showing selected microbial taxa and statistically associated host genes. The component illustrates candidate microbe-host associations related to CRC stage-related biological differences. **(C)** GSVA scores of representative enriched pathways derived from sparse-CCA-associated host gene sets, compared between stage I/II and stage III/IV CRC samples. **(D)** Patient-level Mantel analysis showing associations between microbial and host transcriptomic profiles, with significance assessed by patient-level permutation testing. **(E)** Heatmap summarizing the activity patterns of enriched biological pathways across stage I/II and stage III/IV CRC samples.

A representative sparse-CCA component further illustrated the association between stage-associated microbial taxa and host genes, with the associated gene set involving biological processes related to cell adhesion, developmental regulation, neural-like signaling, and tumor microenvironment remodeling (Figure 4B). Among the top-enriched signaling pathways in this component pair, we compared pathway activities between stage I/II and stage III/IV CRC. Interestingly, these pathways showed distinct activity patterns across clinical stages, with immune- and mucosal-function-related pathways relatively enriched in early-stage CRC and metabolism-related pathways showing higher activity in advanced-stage CRC (Figure 4C). These findings were consistent with stage-associated differences in microbial-host transcriptional programs.

To further examine the global correspondence between microbial and host transcriptomic profiles, we performed a patient-level Mantel analysis using microbial and transcriptomic distance matrices calculated after averaging regional measurements within each patient (Figure 4D). Finally, the pathway activity heatmap summarized the differential activity of these stage-associated functional programs across early- and advanced-stage CRC samples, indicating coordinated stage-associated variation in microbial features and host transcriptional programs (Figure 4E).

### 3.5 Microbe-host integration identifies stage-associated features with potential clinical relevance in CRC

To identify microbe-host association features linked to CRC stage and tissue-level biological stratification, we performed exploratory rCCA at the regional-specimen level using matched microbial and host gene-expression profiles to assess correlations between differential microbial taxa and differentially expressed host genes, using a correlation threshold of 0.6 to select strong microbe-gene association pairs. At the genus level, 10 microbe-host gene association pairs meeting this threshold were identified, mainly linking stage-associated genera with host genes such as MDH1, CD274, FOXC2, GLI4, and CHMP4B (Figure 5A). Clinical relevance analysis using the TCGA CRC cohort showed that the gene signature derived from these host genes was significantly associated with overall survival and that these genes displayed different expression patterns between early- and advanced-stage CRC in TCGA (Figures 5B, C). These results identified candidate links between stage-associated genera and host transcriptional features with potential relevance to CRC stage and patient outcome.

**Figure 5:**
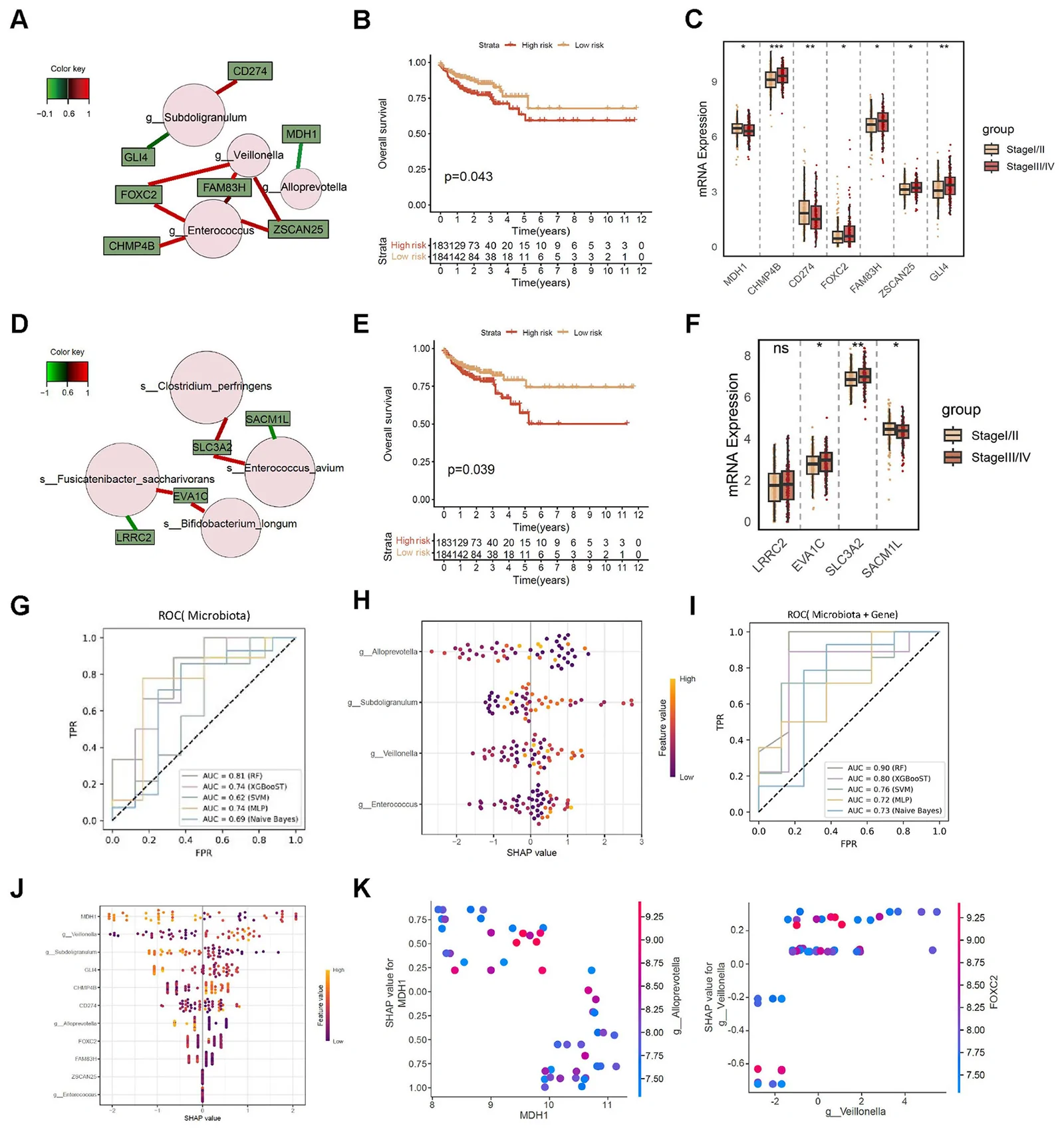
**(A)** rCCA network showing candidate genus-level microbiota-host gene association pairs retained using an absolute correlation threshold > 0.6. Red and green lines indicate positive and negative correlations, respectively. **(B)** Kaplan-Meier survival analysis of the genus-associated host gene signature, including MDH1, CHMP4B, CD274, FOXC2, FAM83H, ZSCAN25, and GLI4, in the TCGA CRC cohort. **(C)** Expression patterns of the genus-associated host genes shown in panel A between stage I/II and stage III/IV CRC patients in the TCGA cohort. **(D)** rCCA network showing candidate species-level microbiota-host gene association pairs retained using an absolute correlation threshold > 0.6. **(E)** Kaplan-Meier survival analysis of the species-associated host gene signature, including SACM1L, SLC3A2, EVA1C, and LRRC2, in the TCGA CRC cohort. **(F)** Expression patterns of the species-associated host genes shown in panel D between stage I/II and stage III/IV CRC patients in the TCGA cohort. **(G)** Receiver operating characteristic (ROC) curves showing the performance of machine-learning models based on core genus-level microbial features for distinguishing stage I/II from stage III/IV CRC. **(H)** SHAP value analysis showing the contribution of each core genus to the XGBoost model. *Alloprevotella* contributed most strongly to the stage-discriminative model, followed by other stage-associated genera. **(I)** ROC curves of combined microbe-gene classifiers integrating core microbial genera and host gene features. **(J)** SHAP value analysis showing feature contributions in the combined microbe-gene XGBoost model. **(K)** SHAP interaction analysis showing representative interaction effects between microbial and host gene features in the combined model.

At the species level, rCCA also identified multiple candidate microbe-host gene association pairs, further supporting potential associations between specific differential taxa and host transcriptional programs (Figure 5D). The corresponding host gene signature was also associated with overall survival in the TCGA CRC cohort and showed differential expression between early- and advanced-stage CRC (Figures 5E, F). Binary classification models based on genus- and species-level associated genes were both able to distinguish stage I/II from stage III/IV CRC in TCGA, suggesting the potential stage-related relevance of these microbiota-associated host genes (Supplementary Figures S3A, B).

Considering the better comparability and stability of genus-level microbial features across datasets, we next constructed stage-classification models based on the core genera. When only core genera were used, multiple machine-learning algorithms showed moderate discriminatory performance, with the highest AUROC reaching approximately 0.81 (Figure 5G). SHAP analysis showed that *Alloprevotella* had the greatest contribution to predictions made by the microbiota-only model, followed by several other stage-associated genera, suggesting that these genera represent important microbial features for distinguishing CRC stage within the model (Figure 5H). To evaluate the generalizability of the core-genus model, we performed external validation using two independent public microbiome datasets, Zeller et al. (2014) and Wirbel et al. (2019). The core genera retained stage-discriminatory ability in both datasets, and the major contributing taxa showed relatively consistent model-explanation weights, supporting the cross-cohort reproducibility of this microbial feature set (Supplementary Figures S3C–F).

On this basis, we integrated core genera with host gene features to construct a combined microbe-gene prediction model. Compared with the genus-only model, the combined model showed markedly improved classification performance, with the highest AUROC increasing from approximately 0.81 to above 0.90, indicating that host transcriptional information substantially enhanced the ability of microbial features to capture CRC stage-associated biological differences (Figure 5I). SHAP analysis of the combined model showed that MDH1 ranked as the top contributing feature, while several genus-level microbial features also retained high explanatory weights (Figure 5J). SHAP interaction analysis further identified non-additive contribution patterns between representative microbial features and host gene features, suggesting that the model's stage-discriminatory performance reflected combined microbial and host transcriptional patterns rather than a single feature (Figure 5K).

Overall, these results show that integrating intratumoral microbiota with host transcriptomic features improves the characterization of biological differences between early- and advanced-stage CRC. The improved performance of the combined microbe-gene model supports coordinated stage-associated variation between microbial and host transcriptional features and identifies candidate signatures for exploratory biological stratification, survival-related investigation, and future mechanistic studies.

### 3.6 The *Alloprevotella*-MDH1 association represents a candidate microbe-host link related to metabolic and immune programs in CRC

In the above analyses, we found that *Alloprevotella* and MDH1 ranked as the top contributing features in the microbiota-only model and the combined microbe-host gene model, respectively. Notably, rCCA analysis also identified *Alloprevotella*-MDH1 as a candidate microbe-host gene association pair. Based on these results, we selected *Alloprevotella*-MDH1 as a representative microbe-host gene pair for further investigation, aiming to assess its relationship with CRC stage, survival-associated features, and relevant biological programs.

Correlation analysis showed that *Alloprevotella* abundance was significantly negatively correlated with MDH1 expression (Figure 6A). This inverse association remained significant at the patient level after averaging regional measurements within each patient and persisted after adjustment for disease stage (Supplementary Table 3). Survival analysis in the TCGA cohort further showed that patients with low MDH1 expression had poorer overall survival, whereas those with high MDH1 expression had relatively better outcomes (Figure 6B), suggesting that MDH1 expression is associated with overall survival in CRC.

**Figure 6:**
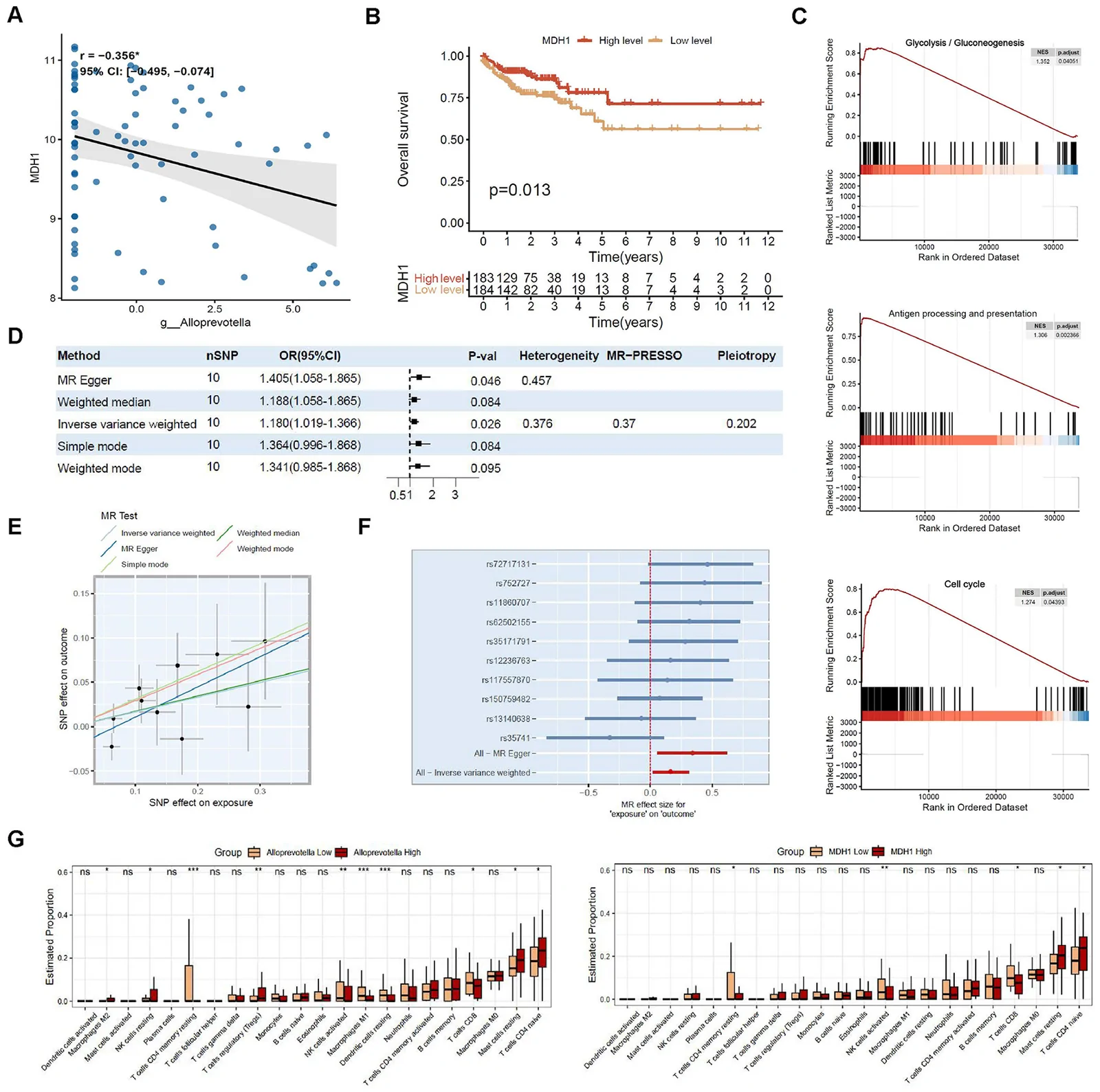
**(A)** Correlation analysis showing the association between *Alloprevotella* abundance and MDH1 expression in CRC tumor samples. **(B)** Kaplan-Meier survival analysis of MDH1 expression in the TCGA CRC cohort. Patients were divided into MDH1-high and MDH1-low groups according to the median expression level. **(C)** GSEA showing representative biological pathways associated with MDH1 expression in CRC. **(D)** Forest plot and summary estimates from forward Mendelian randomization analysis evaluating the genetically predicted association between *Alloprevotella* abundance and CRC risk. **(E, F)** Sensitivity analyses for Mendelian randomization, including scatter plot of MR estimates and forest plot of individual SNP effects related to *Alloprevotella*. **(G)** Immune infiltration analysis assessing whether the *Alloprevotella*-MDH1 association corresponds to distinct immune ecological states in CRC. Samples were stratified according to *Alloprevotella* abundance and MDH1 expression, and inferred immune cell proportions were compared between groups.

MDH1 is a key metabolic enzyme involved in malate-oxaloacetate conversion and the maintenance of cellular redox balance, and is closely related to glucose metabolism and tumor metabolic adaptation (Hanse et al., 2017). To further characterize the biological programs associated with MDH1 in CRC, we performed GSEA analysis. The results showed that MDH1-associated expression patterns were linked to pathways including antigen processing and presentation, glycolysis/gluconeogenesis, and cell cycle regulation (Figure 6C). These findings suggest that MDH1 may not only reflect tumor metabolic status in CRC, but may also be associated with immune recognition and proliferation-related programs. Together with the patient-level inverse association between *Alloprevotella* and MDH1, these results suggest that this microbe-gene association may reflect coordinated variation in intratumoral microbial features, metabolic programs, and immune-related states in CRC.

Considering that *Alloprevotella* is an oral-associated taxon, and that ectopic colonization of oral microorganisms in the intestine and tumor tissues has been recognized as an important feature of CRC microecological remodeling (Downes et al., 2013; Komiya et al., 2019), we further performed Mendelian randomization analysis to explore a potential causal association between *Alloprevotella* and CRC risk. Forward MR analysis showed a positive association trend between *Alloprevotella* and CRC risk, with both MR-Egger and IVW methods indicating the same risk-increasing direction (Figure 6D). Sensitivity analyses provided no strong evidence of heterogeneity or directional horizontal pleiotropy (Figures 6E, F). These findings further support the value of *Alloprevotella* as a CRC-associated microbial feature from a genetic epidemiological perspective, and suggest that ectopic enrichment of oral-associated bacteria may be linked to CRC microecological remodeling and tumor progression, potentially involving MDH1-related metabolic-immune programs.

To further assess whether the *Alloprevotella*-MDH1 association corresponds to a specific immune ecological state in CRC, we compared estimated immune infiltration profiles according to *Alloprevotella* abundance and MDH1 expression levels. The results showed that both grouping strategies distinguished different immune cell composition patterns, suggesting that the *Alloprevotella*-MDH1 association corresponds to variation in the local immune microenvironment (Figure 6G). Together with the aforementioned MDH1-related pathway analysis, these findings suggest that this microbe-gene association may reflect coordinated alterations among intratumoral microbiota, host metabolic programs, and immune ecological states.

Overall, this study identified coordinated stage-associated differences in intratumoral microbiota and host transcriptional programs between early- and advanced-stage CRC. By integrating microbiome profiling, transcriptomic analysis, and machine-learning interpretation, we identified stage-associated microbial features and microbe-host gene associations with potential biological and clinical relevance. The Alloprevotella-MDH1 association provides a representative example of these microbe-host links, supporting the potential value of intratumoral microbe-host features for biological stratification, survival-related interpretation, and future mechanism-oriented studies.

## 4 Discussion

Previous research on the gut microbiome in CRC has predominantly relied on fecal samples, with the primary aim of identifying non-invasive screening tools and early diagnostic biomarkers (Yu et al., 2017a; Zeller et al., 2014). In contrast, the microbiota residing directly within tumor tissue are embedded in the local microenvironment. Their community characteristics may therefore provide complementary information regarding the ecological, metabolic, and immune states of the tumor. In recent years, the intratumoral microbiota has been increasingly recognized as an integral component of the tumor microenvironment, and its distribution is non-random, being tightly coupled with the local ecological state of the tumor (Galeano Niño et al., 2022; Long et al., 2024). Through multi-region sampling, 16S rRNA gene sequencing, and integrated host transcriptomic analysis, the present study identified stage-associated differences in intratumoral microbial composition, diversity, ecological organization, predicted functional potential, and microbiota-host relationships. The patient-level analyses further supported the overall differences in microbial community structure, while the integration of microbial and host transcriptional features revealed complementary signals associated with CRC stage.

At the microbiome level, the intratumoral bacterial communities of stage I/II and stage III/IV CRC showed discernible differences in community composition, diversity indices, co-occurrence patterns, and functional predictions. Samples from advanced-stage tumors exhibited higher microbial diversity, while the co-occurrence networks provided descriptive views of microbial association patterns in the two stage groups. These exploratory findings are consistent with differences in intratumoral microbial ecological organization between the two stage groups. Based on existing knowledge, differences in epithelial integrity, oxygen availability, nutrient conditions, and immune selection between early- and advanced-stage tumors may contribute to the observed microbial patterns (Dejea et al., 2014; Vito et al., 2020), thereby facilitating the colonization of facultative anaerobes and opportunistic pathogens and altering their co-occurrence patterns. The enrichment of oral-associated bacteria, anaerobes, and opportunistic pathogens observed in advanced-stage tumors may therefore reflect changes in nutrient availability, oxygen tension, epithelial integrity, and immune selection within the local tumor niche. The corresponding differences in predicted microbial pathways further suggest that these compositional changes are accompanied by alterations in microbial metabolic and signaling potential. Together, the diversity, co-occurrence, and functional findings support the presence of distinct intratumoral microbial ecological states across CRC stages.

More importantly, these microbial alterations do not occur in isolation. Patient-level Procrustes and Mantel analyses, together with regional-level canonical correlation analyses, identified coordinated associations between microbial community structure and host gene expression. Because the transcriptome of tumor tissue represents an integrated output of tumor cells, immune cells, stromal cells, and vascular endothelial cells, this cross-omics coupling may reflect coordinated shifts among the local microecology, inflammatory responses, immune regulation, metabolic adaptation, and stromal remodeling. Previous studies focusing on individual bacterial species, such as Fusobacterium nucleatum (Kostic et al., 2013; Mima et al., 2016; Yu et al., 2017b), have demonstrated that specific microorganisms can influence tumor behavior by activating immune evasion pathways or altering local metabolite concentrations. Recent integrative analysis of CRC tissues has similarly identified extensive associations between intratumoral bacterial taxa and host transcriptional programs and has further shown, through experimental validation of selected microorganisms, that individual bacteria may influence immune and tumor-related pathways. These findings support a biological framework in which intratumoral microorganisms and host cellular programs form interconnected ecological and molecular networks. The sparse-CCA and rCCA results in our study provided candidate links between microbial taxa and host gene modules related to metabolism, immune and mucosal functions, cell adhesion, cell-cycle regulation, and tumor-associated signaling. This coordinated pattern is also consistent with recent reviews describing bidirectional microbiota-cancer crosstalk and emphasizing the value of multi-omics analysis for resolving complex interactions between microbial communities and host tumor states (Moshref-Javadi et al., 2026).

Through multi-omics integration and machine-learning, we further identified a set of staging-associated microecological and host gene joint signatures. A classification model constructed using only core genera showed modest performance, whereas the addition of host gene information substantially improved stage discrimination. Given the limited cohort size, these models should be interpreted as exploratory stage-discrimination models rather than clinically validated predictive tools. Single-omics models in oncology research often suffer from insufficient stability. This limitation is particularly relevant in CRC, where tumor heterogeneity, sample-processing variation, and context-dependent host-microbiota interactions can weaken the reproducibility of signatures derived from a single data layer (Aran et al., 2015; Eisenhofer et al., 2019). Our results indicate that microbial and host gene signatures carry complementary rather than redundant information. The microbiota reflects the degree of local ecological openness, whereas host genes reflect the cellular response programs to those ecological changes. When combined, these complementary features may allow the model to more comprehensively capture the biological differences between early- and advanced-stage CRC. Simultaneous assessment of genus-level microbial abundance and selected host gene expression in formalin-fixed paraffin-embedded tissues or core needle biopsies is technically feasible and provides a potential basis for future clinical translation. With further validation, such a dual-detection approach may help identify tumors with biologically aggressive features that are not fully represented by anatomical staging, thereby complementing the current TNM classification system. The reproducibility of the core microbial features in external microbiome datasets further suggests that part of the identified microbial signal is shared across independent CRC cohorts. Recent computational pathology research has likewise reported that tumor architectural features associated with barrier disruption, invasion, anatomical site, and stage may capture aspects of microbiome-permissive tissue states (Steele et al., 2026). These findings provide complementary evidence that microbial ecology is closely related to the structural and molecular context of CRC tissues and may contribute additional biological information beyond conventional pathological descriptors.

Among the many associated features, the pairing of *Alloprevotella* and MDH1 is the most representative. *Alloprevotella* was the top contributor in the genus-only model, while MDH1 ranked highest in the combined model, with the two showing an inverse association in regional-level regularized canonical correlation analysis that remained evident in the patient-level sensitivity analysis. Their consistent identification across correlation and model-interpretation analyses led us to select *Alloprevotella*-MDH1 as a representative microbe–host association. *Alloprevotella* is an oral commensal bacterium, and previous epidemiological studies have suggested a link between oral hygiene status and CRC risk, though the evidence has largely remained at the population survey level. The presence of an oral-associated taxon among the major intratumoral microbial features is consistent with increasing evidence that microorganisms originating from the oral cavity may occupy intestinal and tumor-associated ecological niches in CRC. Higher *Alloprevotella* abundance was associated with lower MDH1 expression, and low MDH1 expression was itself associated with poor prognosis. MDH1, a key enzyme in the interconversion of malate and oxaloacetate, directly influences cellular redox balance and glycolytic flux, a topic that has long attracted attention in the context of tumor metabolic reprogramming (Hanse et al., 2017; Mansouri et al., 2017; Leverett et al., 2024). Gene set enrichment analysis further linked MDH1 to antigen processing and presentation, glycolysis, and cell cycle pathways, suggesting that shifts in metabolic state may concurrently affect immune recognition and proliferation control. The corresponding differences in estimated immune-cell composition further connect this microbe-host association with the local immune context of CRC. Collectively, these observations position the *Alloprevotella*-MDH1 pair at the intersection of intratumoral microbial ecology, host metabolism, and immune-related transcriptional programs. The Mendelian randomization findings provide additional genetic epidemiological support for an association between *Alloprevotella* and CRC susceptibility, adding another layer of evidence to the tissue-based and transcriptomic observations.

These findings also have potential implications for the developing field of microbiome-informed oncology. Microbial signatures, host transcriptional programs, and tumor architectural characteristics may eventually provide complementary information for biological stratification within conventional clinicopathological categories. Rather than relying on a single taxon or host gene, integrated microbe-host signatures may better represent the ecological and molecular heterogeneity of CRC. Recent experimental studies in other tumor types have shown that direct manipulation of intratumoral bacteria can alter tumor-associated biological processes, illustrating the potential value of targeting selected microbial components in appropriately validated settings (Wang et al., 2026). Broader oncology literature has also highlighted the possible contribution of the microbiota to antitumor immunity and immunotherapy responses (Mattavelli et al., 2026). In this context, the microbial taxa, host genes, and microbe-host pairs identified in the present study provide candidates for future biomarker and mechanistic studies aimed at determining whether tumor-associated microbial ecology can complement established pathological and molecular classification systems.

Within the current TNM staging system, anatomical stage remains the cornerstone for determining the extent of surgery and adjuvant treatment strategies. Yet, clinically, patients at the same stage can exhibit marked prognostic heterogeneity, with the risk of recurrence or metastasis in some cases far exceeding the average for that stage (Dienstmann et al., 2017b; Pagès et al., 2018), indicating that lymph node and distant metastasis status alone cannot fully capture the biological aggressiveness of a tumor. Our microbiome-host integrative framework adds a molecular-ecological dimension to anatomical staging. The combined characterization of microbial communities and host transcriptional programs may improve the recognition of biological heterogeneity among tumors assigned to the same anatomical stage. This framework is not intended to replace TNM staging but may provide a basis for developing complementary biological stratification approaches within the existing clinical classification system.

It must be acknowledged, however, that the relatively small, single-center cohort may limit the generalizability of the findings, particularly for individual microbial taxa and anatomical subsites. Moreover, because distant metastasis was concentrated in the stage III/IV group, some observed stage-associated differences may partly reflect metastatic status, which could not be fully disentangled in the current cohort. The cross-sectional design also precludes determination of temporal changes during CRC progression and the direction of the observed microbe-host associations. Moreover, because several analyses included multiple regional specimens from the same patient, residual within-patient dependence may influence regional-level exploratory results, although patient-level aggregation or patient-aware models were used where applicable. In addition, 16S rRNA sequencing provides limited species- and strain-level resolution and does not directly measure microbial activity. Although procedural blanks indicated low background contamination, the absence of sequenced negative controls precluded sequence-level contaminant identification; however, identical processing across groups makes systematic stage-related bias less likely. All patients received the same perioperative antibiotic prophylaxis approximately 30 min before surgery, and tissue was collected immediately after resection. The short interval and uniform protocol likely limited its effect on group-level comparisons, although an influence on the intratumoral microbiota cannot be excluded. The co-occurrence networks, predicted microbial functions, pathway enrichment, immune-infiltration estimates, and microbe-host correlations therefore require further validation using multi-omics and experimental approaches. Although the core microbial features showed reproducibility in external microbiome datasets, the complete combined microbe-gene model requires validation in larger independent cohorts with matched microbiome and transcriptome data. Further functional studies are also needed to determine the biological basis of the *Alloprevotella*-MDH1 association.

In summary, this study identified coordinated differences in intratumoral microbial ecology and host transcriptional programs between early- and advanced-stage CRC. Through multi-omics integration, this study identified a set of stage-associated microecological and host joint signatures and, using the *Alloprevotella*-MDH1 association as an illustrative example, provided a preliminary view of their connections at the metabolic and immunological levels. These findings broaden our understanding of the relationship between intratumoral microbial communities and host molecular states in CRC. Subject to replication in external cohorts and prospective study designs, they may accumulate more robust evidence for biological stratification and prognostic evaluation based on the interplay between the microbiota and the host.

## Data Availability

The raw sequence data (RNA and bacteria) generated in this study have been deposited in the GSA-human Sequence Read Archive (SRA) database under accession number: PRJCA058722. Any additional information required to reanalyze the data reported in this paper is available from the lead contact upon request.
