# Supplementary material for: Intratumoral microbiota-host gene associations characterize stage-associated microecological patterns in colorectal cancer
Source: Front Microbiol. 2026 Sep 28;17:1934580. doi: 10.3389/fmicb.2026.1934580 (PMC13654950; doi:10.3389/fmicb.2026.1934580)
Supplement: Supplementary file 1 [file Supplementary_File_1.docx]

Figure S1

Absolute standardized mean differences (SMDs) were calculated for age, sex, body mass index, tumor size, primary tumor laterality, primary tumor site, smoking history, alcohol consumption, and distant metastatic status among 30 independent patients.

Figure S2

(A) Volcano plot showing differentially expressed genes between stage I/II and stage III/IV CRC tumor samples.

(B) PCoA plot based on host gene-expression profiles showing transcriptomic separation between stage I/II and stage III/IV CRC samples.

(C) Three-dimensional principal component analysis showing global variation in host gene-expression profiles across CRC stages.

(D) Patient-level Procrustes analysis showing concordance between intratumoral microbial and host transcriptomic profiles, with significance assessed by patient-level permutation testing.

Figure S3

(A) ROC curves of binary-classification models constructed using genus-level rCCA-associated host genes to distinguish stage I/II from stage III/IV CRC in the TCGA CRC cohort.

(B) ROC curves of binary-classification models constructed using species-level rCCA-associated host genes to distinguish stage I/II from stage III/IV CRC in the TCGA CRC cohort.

(C,D) External validation of the core genus signature in the ZellerG_2014 public CRC microbiome cohort, shown by ROC curves and SHAP value analysis.

(E,F) External validation of the core genus signature in the WirbelJ_2018 public CRC microbiome cohort, shown by ROC curves and SHAP value analysis.

Supplementary Table 1

| **Patient number** | **TNM Stage** | **Sex** | **Age** | **BMI** | **Tumor laterality** | **Size** | **Location** | **TNM** | **Lymph node metastasis** | **Distant metastasis** | **Smoke** | **Alcohol** |
| --- | --- | --- | --- | --- | --- | --- | --- | --- | --- | --- | --- | --- |
| **1** | **I** | female | 64 | 29.7 | Left | <5cm | Sigmoid colon | T1N0M0 | no | no | no | no |
| **2** | **I** | female | 70 | 24.1 | Left | <5cm | Sigmoid colon | T2N0M0 | no | no | no | no |
| **3** | **I** | male | 69 | 18.4 | Left | <5cm | Rectum, 10 cm from the anal verge | T1N0M0 | no | no | no | yes |
| **4** | **II** | male | 73 | 27.7 | Left | <5cm | Sigmoid colon | T3N0M0 | no | no | yes | no |
| **5** | **II** | male | 50 | 25.4 | Right | >5cm | Ileocecal region | T4N0M0 | no | no | no | no |
| **6** | **II** | female | 44 | 27 | Right | >5cm | Ascending colon | T3N0M0 | no | no | no | no |
| **7** | **II** | male | 57 | 21.7 | Right | <5cm | Ascending colon | T3N0M0 | no | no | no | no |
| **8** | **II** | male | 84 | 21.5 | Left | >5cm | Rectum, 5 cm from the anal verge | T3N0M0 | no | no | no | no |
| **9** | **II** | male | 68 | 20.4 | Left | <5cm | Rectum, 9 cm from the anal verge | T4N0M0 | no | no | no | no |
| **10** | **II** | male | 61 | 22.4 | Right | >5cm | Ascending colon | T4N0M0 | no | no | no | no |
| **11** | **II** | male | 73 | 21.5 | Left | <5cm | Rectum, 15–19 cm from the anal verge | T3N0M0 | no | no | no | no |
| **12** | **II** | male | 41 | 19.2 | Right | <5cm | Transverse colon | T4N0M0 | no | no | no | no |
| **13** | **II** | male | 65 | 20.2 | Left | <5cm | Descending colon | T3N0M0 | no | no | yes | no |
| **14** | **II** | female | 70 | 18.3 | Right | <5cm | Transverse colon | T3N0M0 | no | no | no | no |
| **15** | **III** | female | 43 | 22.2 | Right | <5cm | Ascending colon | T4N1M0 | yes | no | no | no |
| **16** | **III** | female | 38 | 25.3 | Left | <5cm | Rectum, 10–15 cm from the anal verge | T4N2M0 | yes | no | no | no |
| **17** | **III** | male | 83 | 25.4 | Left | <5cm | Sigmoid colon | T3N1M0 | yes | no | no | no |
| **18** | **III** | male | 66 | 24.2 | Left | <5cm | Sigmoid colon | T2N1M0 | yes | no | yes | no |
| **19** | **III** | male | 41 | 26 | Right | <5cm | Ileocecal region | T4N1M0 | yes | no | no | no |
| **20** | **III** | male | 66 | 24.6 | Left | <5cm | Rectosigmoid junction | T4N2M0 | yes | no | no | no |
| **21** | **III** | male | 82 | 20.2 | Left | >5cm | Sigmoid colon | T4N1M0 | yes | no | no | no |
| **22** | **III** | male | 70 | 25.1 | Right | >5cm | Ileocecal region | T3N1M0 | yes | no | no | no |
| **23** | **III** | male | 69 | 22.7 | Right | <5cm | Ascending colon | T4N2M0 | yes | no | no | no |
| **24** | **III** | male | 65 | 23.3 | Left | <5cm | Rectum, 16–19 cm from the anal verge | T4N1M0 | yes | no | no | no |
| **25** | **III** | male | 69 | 26.7 | Left | <5cm | Sigmoid colon | T4N2M0 | yes | no | yes | no |
| **26** | **IV** | female | 65 | 27.9 | Left | <5cm | Rectum, 12–15 cm from the anal verge | T4N2M1 | yes | yes | no | no |
| **27** | **IV** | female | 65 | 27.1 | Left | >5cm | Descending colon | T4N0M1 | no | yes | no | no |
| **28** | **IV** | male | 73 | 23.8 | Left | >5cm | Sigmoid colon | T3N1M1 | yes | yes | no | no |
| **29** | **IV** | male | 68 | 24.6 | Left | >5cm | Rectum, 12–18 cm from the anal verge | T4N1M1 | yes | yes | no | no |
| **30** | **IV** | male | 63 | 22 | Left | >5cm | Rectum, 5 cm from the anal verge | T3N2M1 | yes | yes | no | no |

**Clinical and pathological characteristics of the study cohort**

Supplementary Table 2

| patient | group | sample | patient | group | sample |
| --- | --- | --- | --- | --- | --- |
| A | CRC I/II | AT1 | R | CRC III/IV | RT1 |
|  |  | AT2 |  |  | RT2 |
|  |  | AT3 |  |  | RT3 |
| B | CRC I/II | BT1 | S | CRC III/IV | ST1 |
|  |  | BT2 |  |  | ST2 |
|  |  | BT3 |  |  | ST3 |
| C | CRC I/II | CT1 | T | CRC III/IV | TT1 |
|  |  | CT2 |  |  | TT2 |
| D | CRC I/II | DT1 |  |  | TT3 |
| E | CRC I/II | ET1 | U | CRC III/IV | UT1 |
|  |  | ET2 |  |  | UT2 |
|  |  | ET3 |  |  | UT3 |
| F | CRC I/II | FT1 | V | CRC III/IV | VT1 |
|  |  | FT2 |  |  | VT2 |
|  |  | FT3 |  |  | VT3 |
| G | CRC I/II | GT1 | W | CRC III/IV | WT1 |
| H | CRC I/II | HT1 |  |  | WT2 |
|  |  | HT2 |  |  | WT3 |
|  |  | HT3 | X | CRC III/IV | XT1 |
| I | CRC I/II | IT1 |  |  | XT2 |
|  |  | IT2 |  |  | XT3 |
|  |  | IT3 | Y | CRC III/IV | YT1 |
| J | CRC I/II | JT1 | Z | CRC III/IV | ZT1 |
| K | CRC I/II | KT1 |  |  | ZT2 |
|  |  | KT2 |  |  | ZT3 |
| L | CRC I/II | LT1 | AA | CRC III/IV | AAT1 |
| M | CRC I/II | MT1 |  |  | AAT2 |
| N | CRC I/II | NT1 |  |  | AAT3 |
|  |  | NT2 | AB | CRC III/IV | ABT1 |
| O | CRC III/IV | OT1 |  |  | ABT2 |
|  |  | OT2 |  |  | ABT3 |
| P | CRC III/IV | PT1 | AC | CRC III/IV | ACT1 |
|  |  | PT2 |  |  | ACT2 |
|  |  | PT3 | AD | CRC III/IV | ADT1 |
| Q | CRC III/IV | QT1 |  |  | ADT2 |
|  |  | QT2 |  |  | ADT3 |
|  |  | QT3 |  |  |  |

**Patient–specimen mapping for the final analysis**

Supplementary Table 3

| Analysis | Estimate | Lower 95%CI | Upper 95%CI | P value | CI method |
| --- | --- | --- | --- | --- | --- |
| Patient-level Spearman correlation | -0.36431 | -0.66256 | -0.003 | 0.047792 | BCa bootstrap |
| Stage-adjusted linear regression | -0.13367 | -0.22519 | -0.04216 | 0.005789 | Model-based |
| Stage-adjusted standardized regression | -0.39821 | -0.67083 | -0.12558 | 0.005789 | Model-based |

**Patient-level sensitivity analyses of the Alloprevotella–MDH1 association**
